# A Systematic Review and Meta-Analysis of Alpha Synuclein Auto-Antibodies in Parkinson's Disease

**DOI:** 10.3389/fneur.2018.00815

**Published:** 2018-10-01

**Authors:** Kirsten M. Scott, Antonina Kouli, Su L. Yeoh, Menna R. Clatworthy, Caroline H. Williams-Gray

**Affiliations:** ^1^Department of Clinical Neurosciences, John van Geest Centre for Brain Repair, University of Cambridge, Cambridge, United Kingdom; ^2^University of Cambridge School of Clinical Medicine, Cambridge, United Kingdom; ^3^MRC Laboratory of Molecular Biology, Molecular Immunity Unit, Department of Medicine, University of Cambridge, Cambridge, United Kingdom

**Keywords:** antibodies (Abs), alpha synuclein (α syn), auto-antibodies, Parkinson's disease (PD), peripheral inflammation, Fcγ receptor

## Abstract

Immune dysfunction has been associated with Parkinson's disease (PD) and its progression. Antibodies play an important role in both innate and adaptive responses, acting as powerful effector molecules that can propagate inflammation by activating innate immune cells. Alpha synuclein binding antibodies have been described in PD patients with conflicting associations. In this article, we consider the potential mechanistic basis of alpha synuclein auto-antibody development and function in PD. We present a systematic review and meta-analysis of antibody studies in PD cohorts showing that there is weak evidence for an increase in alpha synuclein auto-antibodies in PD patients particularly in early disease. The confidence with which this conclusion can be drawn is limited by the heterogeneity of the clinical cohorts used, inclusion of unmatched controls, inadequate power and assay related variability. We have therefore made some recommendations for the design of future studies.

## Introduction

Parkinson's disease (PD) is a neurodegenerative disorder characterized by loss of dopaminergic neurons in the substantia nigra resulting in a movement disorder and many non-motor symptoms, including dementia, postural hypotension and gut dysfunction (1). Whilst dopaminergic treatments may alleviate the motor symptoms, there are currently no disease-modifying therapies that slow clinical progression.

Immune dysfunction has been associated with PD and its progression (2–5) and represents a tractable target for disease modification. However, the cellular and molecular mechanisms underpinning this association have yet to be elucidated. Potential pathways include the activation of adaptive immunity via antigen-specific recognition of alpha-synuclein or non-specific innate immune activation due to cell damage and death.

The accumulation of aggregated alpha-synuclein within CNS neurons is the pathological hallmark of PD (6). There is also evidence that misfolded alpha synuclein accumulates in the periphery, for example in the gut, in early stages of disease (7), providing a route for the exposure of the peripheral immune system to a central nervous system (CNS) antigen. Monomeric alpha synuclein is abundant in the CNS in pre-synaptic terminals of neurons and is also produced by platelets and red blood cells peripherally. Pathological forms of the protein range from soluble oligomers to mature insoluble fibrillar forms (8). Multiple studies have sought to measure alpha synuclein in either the blood or serum [reviewed in (9)] for use as a biomarker. Substantial variation in levels may be a confounding factor in studies measuring alpha synuclein antibodies as these may be undetectable if already bound.

A recent study demonstrated the presence of alpha-synuclein specific CD4 and CD8 T cells in PD patients, implicating an alpha-synuclein specific adaptive immune response in disease pathogenesis (10). CD4 T cells orchestrate adaptive immunity, including humoral responses which result in the production of antibodies. Antibodies are powerful immune effector molecules produced by plasma cells, terminally differentiated B lymphocytes. The most common circulating antibody isotype is IgG, that can readily initiate and propagate inflammation by activating complement and engaging cell surface antibody receptors [Fcγ receptors (FcγR)] that are expressed by most innate immune cells.

Alpha synuclein-specific IgG antibodies have been described in PD, but their role is unclear with many conflicting studies. Publication bias favoring positive findings in this field may also further complicate attempts to unmask a true effect. The presence of alpha synuclein specific antibodies in early disease could potentially contribute to pathology by exacerbating local inflammation in the brain, promoting neuronal damage and causing disease progression. Consistent with this hypothesis, IgG isolated from PD patients and injected into rat substantia nigra causes selective dopaminergic cell death that was absent in animals receiving control IgG (11). There is also attenuation of disease in Fcγ receptor knockout mice receiving PD IgG, confirming that activation of microglia by PD IgG is pathogenic (12). Approximately 30% of dopaminergic cells in the substantia nigra of post-mortem PD brains were bound by IgG highlighting that immunoglobulins do cross the blood brain barrier in PD and may play a role in disease (13).

Alternatively, alpha synuclein auto-antibodies may play a protective role, facilitating the clearance of toxic protein species by opsonizing alpha-synuclein for FcγR-mediated uptake by phagocytes. Consistent with this hypothesis, the passive peripheral transfer of alpha-synuclein specific antibodies in some mouse models of PD improved disease outcomes (14). Trials of both passive and active immunization therapies targeting alpha synuclein are underway (15, 16).

Clearly, it is critical to have a better understanding of how alpha synuclein autoantibodies relate to PD and its progression. In particular, there is a need to discern whether they constitute a useful diagnostic or prognostic biomarker or may have potential therapeutic relevance. In this article, we will consider the potential mechanistic basis of their role in PD, present a systemic review of antibody studies in PD cohorts, critically discuss the value and limitations of existing data and make recommendations for future studies.

## Potential mechanisms underlying antibody generation in PD

**Figure 1:**
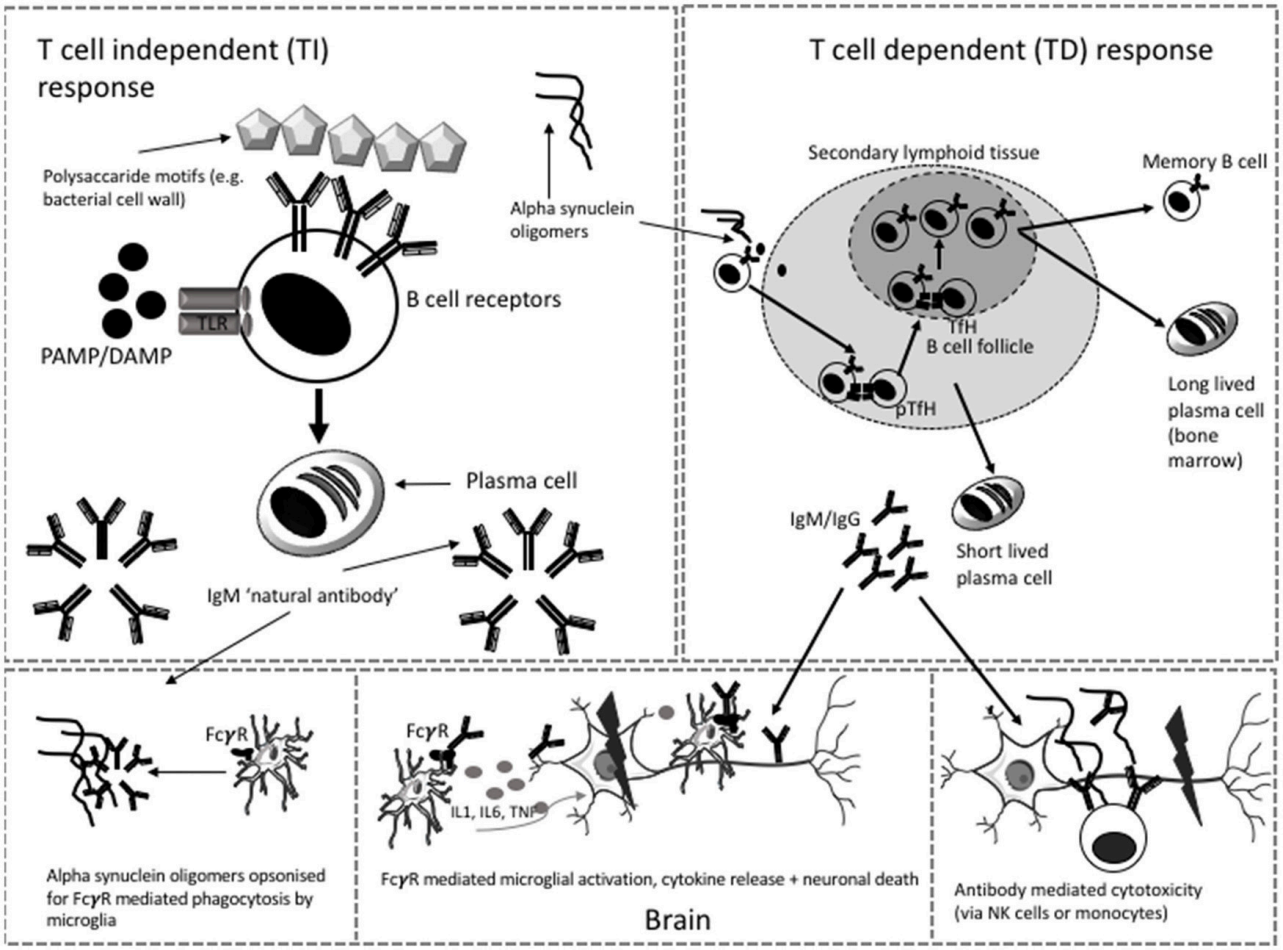
Possible T independent and T dependent mechanisms of antibody generation in PD. Microglia and neuron images modified from templates obtained https://smart.servier.com/smart_image/microglia-3/ under Creative Commons Attribution 3.0 Unported License. TfH, T follicular helper cell; pTfH, peripheral T follicular helper cell; NK, Natural killer; PAMP, pathogen associated molecular motif; DAMP, damage associated molecular motif, TLR, toll like receptor.

B lymphocytes can produce antibodies via T cell-independent (TI) and T cell-dependent pathways (TD) (see Figure 1). TI pathways involve the recognition of multimeric carbohydrate and lipid antigens by the B cell receptor (BCR) or by toll like receptors (TLR) on the cell surface of “B1” cells (or marginal zone B cells in the spleen). This leads to the production of polyreactive IgM that binds with low affinity and can facilitate the removal of blood borne encapsulated organisms (17). Antibodies produced in this context are called “natural antibodies.” Most of the literature on alpha synuclein antibodies suggests that these are natural antibodies (18–21). Natural antibodies are part of innate immune surveillance against pathogens or cell damage and are present from an early point in development (22). They are predominantly IgM but IgG and IgA natural antibodies have also been described (22). Antibodies to alpha synuclein epitopes could be generated via this process.

The recent description of alpha synuclein specific T cells in patients with PD (10) supports the thesis that alpha synuclein antibodies may be generated by a TD response. These antibodies recognize protein antigens and their production requires a cognate interaction between “B2” cells and CD4 T cells. This facilitates iterative rounds of somatic hypermutation and clonal selection within a germinal center reaction to generate class-switched long lived plasma cells or memory B cells capable of initiating a secondary response upon further encounter of the antigen (22). The plasma cells that arise from this process are able to produce large quantities of specific, high affinity class-switched antibodies (17). Humoral responses to self-antigen are limited by negative selection of self-reactive clones during B cell development. However, if the self-antigen is modified sufficiently, as in the case of alpha synuclein toxic species, and is present in an immunogenic context, such as cell death, some B cell clones may be activated to produce alpha synuclein antibodies. Such an antibody response might change over time; firstly IgM may dominate, but with progression of the germinal center reaction, there is class switching to IgG or IgE. Secondly, with persistent exposure to neo-antigen, clones with higher levels of somatic hypermutation and higher affinity antibodies would be selected. Thirdly, the overall level of alpha synuclein antibody might change with age, as older age is associated with decreasing antibody response to antigens (e.g., vaccines) and immunosenescence of the B cell compartment (23).

B cell activation to generate plasma cell-producing antibodies generally occurs within secondary lymphoid organs (lymph nodes and spleen) but may also occur in tertiary lymphoid follicles that develop in inflamed tissues. The site of B cell activation to generate alpha synuclein-specific responses is unclear and may be peripheral or within the CNS. Follicles have been described in the meninges of patients with multiple sclerosis (24), with the potential to generate CNS localized antibodies, but whether such structures exist in PD is unknown.

Antibodies bind non-specifically to Fcγ receptors on other immune cells (e.g., phagocytes, monocytes, dendritic cells) or via engagement of their Fc region with complement components (25). Different subclasses of IgG (IgG1, IgG2, IgG3 and IgG4) have different affinities for the FcγR on cells which can be either activating or inhibitory [see (25)]. There is evidence that FcγRI and FcγRIIB/C are required for uptake of alpha synuclein by CNS derived cells in culture and that this is mediated by the presence of alpha synuclein specific antibodies (26). One recent paper suggested that FcγRIIB (a low affinity inhibitory Fc receptor) is not only responsible for the inhibition of phagocytosis of alpha synuclein fibrils (via low affinity binding with the fibrils themselves) but also mediates cell to cell transmission of alpha synuclein (27). In addition, the glycosylation status of immunoglobulin also affects downstream binding and effector function (28). A study investigating the IgG glycome in PD showed significant differences between patients and controls, with the authors concluding that the changes observed in PD may result in enhanced Fcγ RIIIa-mediated antibody-dependent cellular cytotoxity (with the potential to contribute to chronic inflammation) (29).

## Systematic review of alpha synuclein antibody studies in PD

We [AK, SLY and KS] searched the literature for studies published prior to 1st June 2018 using Pubmed, Medline, Cochrane database, Embase, Google scholar and Keele Web of Science. We used the following search terms: “Antibody and Parkinson's Disease,” “Auto-antibody and Parkinson's disease,” “Alpha synuclein antibody,” “Alpha synuclein auto-antibody.” To ensure complete study capture we also searched using “Auto-antibody dementia” “Antibody dementia.” Reference lists of the selected papers were also manually searched to identify additional studies. Papers were excluded if they did not involve PD patients, if they did not measure alpha synuclein antibodies and if there was no control group. Otherwise all papers measuring antibodies to alpha synuclein or its epitopes in Parkinson's disease patients were included in the systematic review. The literature searches were done between 1 May 2018 and 6 June 2018. Summary information from each study was compiled into a table (Table 1).

**Table 1:** Summary of studies measuring alpha synuclein antibodies in Parkinson's disease.

	**Paper**	**Method**	**Fluid**	**N (HC = healthy controls)**	**Matched**	**Mean age of PD (SD)**	**Disease duration years (SD)**	**H and Y (SD)**	**Finding (in PD vs. controls)**	**Required N^∧∧^**
< = 5 years DD	Xu et al. (30)^***^	Electrochemical impedance spectroscopy	Serum	60 PD, 29 HC	Yes	69.4 (SD10.8)	1.4 (1.44)	20 HandY1, 20 HandY2, 20 HandY3	↑ in PD, more in HandY 1 and 2 than controls, no diff betweeen stages	382
	Horvath et al. (31)^***^	indirect ELISA	Plasma, CSF	20 PD, 20 HC	Yes	Mild: 65.5 (38–79^*^) Moderate: 67.2 (56–77^*^)	2.8 (1–8^*^) months (< 1 year)	1.5 to 2	↑ in PD vs. HC in CSF and plasma Decreased in moderate vs. mild disease	N/A
	Smith et al. (9)	ELISA	Serum	14 PD, 11 PD syndrome, RBD 10, 9 HC	Yes	RBD 58 (SD 9), PD 63 (9)	Median 3.5 (1–12^*^)	1.3 (range 1–3.5)	No difference	N/A
	Gruden et al. (32)^***^	ELISA	Serum	32 PD, 26 HC	Yes	60.8 (2)^**^	8.6 (3.4)^**^ Subgroup < 5	2.1 (0.6)	↑ in PD vs. HC, greater difference with monomers than oligomers	23
	Shalash et al. (33)	ELISA	Serum	46 PD, 20 HC	Yes	56.26 (SD12.26)	5.2 (3.36)	3 (1.5–3.5 range)	↑ in PD vs. HC	N/A
7-10 years DD	Akhtar et al. (34)	ELISA	Serum, CSF	Serum: 53 PD, 16 HC CSF: 93 PD, 52 HC Both CSF and serum for 24 participants	No	Serum 70.9 (7) CSF 67.1 (9.4)	7.9 (5)	3 (1–4) (median + range)	CSF ↑, serum →	77
	Brudek et al. (19)	ELISA, MSD	Plasma	46 iPD, 46 HC	No	62.4 (6.7)	7.9 (5)	2 (median)	↓ in PD vs. HC	126
	Papachroni et al. (35)	Immunoblot	Serum	31 iPD, 20 FPD, 26 HC	Yes	Idiopathic: 65.1 (11.6), Familiial: 66.1 (12.7)	Calculated 7.2 iPD, 9.4 FPD	2.4 (FPD), 2.5 iPD	↑ in FPD vs. PD or controls	N/A
	Yanamandra et al. (36)^***^	Elisa, western blot, biocore surface plasmon resonance	Serum	39 PD, 23 HC	Yes	55.7 (10)	7.7 (5.6)	HandY1-2 27, HandY 2.5–4 12	↑ PD vs. HC	N/A
	Caggiu et al. (37)	ELISA	Serum	40 PD, 40 HC	Yes	69.8 (7.95)	8.42(4.29)	3.01 (0.88)	↑ in PD vs. controls to three peptides (similar to HSV)	N/A
	Maetzler et al., (38)	ELISA	Serum	93 PD (demented subgroup 31), 194 controls	No	68.5(SD9) PDND, 76.7 (SD8) PDD	9.5 (1–26^*^)	2 (1–4)	No difference	N/A
10-12 years DD	Alvarez-Castelao et al. (39)	ELISA immunoblots	Plasma	55 iPD, 104 LRRK2 carriers, 85 HC	No	67.8 (9.9) iPD 68.37 (10.2) LRRK2	12 (8.7) iPD 13 (11) LRRK2	2.44 (0.8) iPD, 2.55 (0.88) LRRK2	Controls and iPD no difference Using stringent criteria ↑ antibodies in LRKK2 pre-manifest	N/A
	Besong-Agbo et al. (18)	ELISA	Serum	62 iPD, 46 HC	Yes	68.6 (9)	10.2(6)	>3	↓ in PD vs. HC	60
Unknown DD	Bryan et al. (40)	Electrochemical impedance spectroscopy	Serum	30 PD, 14 HC	No	Not reported	Not reported	1 to 3	↑ in PD vs. HC, increasing up to HandY 2 then decreasing for HandY 3.	N/A
	Heinzel et al. (20)	ELISA	Serum, CSF	66 PD, 69 HC (CSF 59 PD and 46 controls)	Yes	No ages reported	Not reported	2	No difference	214
	Woulfe et al. (41)	ELISA	Serum	Serum: 28 PD, 19 HC CSF: 4 PD, five controls	Not reported	Not reported	Not reported	Not reported	No difference	N/A

*Numbers refer to mean and standard deviation unless otherwise specified. The table is ordered according to disease duration. Patient and control groups were considered “matched” if there were no significant between-group differences in age and gender distributions. One paper was removed due to cohort overlap (42). N/A was recorded in the sample size column if there was not sufficient data to do the power calculation*.

* *Range*,

** *SEM*,

*** *author overlap*,

∧∧ *in each group, DD, Disease duration*.

In order to assess whether studies were adequately powered, mean alpha synuclein antibody titres (or optical density) in each group and standard deviations were recorded and used to calculate required sample size to detect a difference of the magnitude reported. The following formula was used to calculate sample size [modified from (43)].

$$\begin{array}{l} nA=\kappa nB\;and\;nB=\left(1+\frac{1}{\kappa }\right){\left(Swithin\frac{z1-\frac{a}{2\;}+z1-\beta }{\mu A-\mu B}\;\right)}^{2} \end{array}$$

Where:

K = nA/nB (matching ratio between groups—nA = PD patients, nB = controls)

Swithin = pooled standard deviation across groups

α = Type I error (set at 0.05)

β = Type II error (1-β = power, set at 0.8)

The pooled within sample standard deviation was calculated to overcome differences in variation between the groups [from (44)]:

$$\begin{array}{l} Swithin=\;\sqrt{\frac{\left(n1-1\right){S1}^{2}+\left(n2-1\right){S2}^{2}}{n1+n2-2}} \end{array}$$

n1 = sample size (SS) in patients, n2 = SS in controls

S1 = SD in patients, S2 = SD in controls.

## Meta-analysis of alpha synuclein antibody studies in PD

We undertook a meta-analysis, stratified by disease duration given the suggestion in the literature that this is a relevant factor [e.g., (36)]. Studies with mean disease durations of 5.9 years and less were included in an “early disease” meta-analysis and those with disease durations of 7 years or more were included in a “later disease” meta-analysis given the trends noted in the review above and in Table 1.

More stringent data quality criteria were adopted for the meta-analysis than for the systematic review described above.

Inclusion criteria:

- The study measured antibodies to full length alpha synuclein
- The antibodies were measured using titres (either relative or absolute) as a continuous measure
- The study included both idiopathic PD patients and controls
- The study stipulated a measure of disease duration for the cohort
- The controls were age and gender matched to the patients
- Antibodies were measured in either serum or plasma

If a study had not published appropriate statistical tests to determine whether the controls were matched appropriately to the patients this was performed (independent samples *t*-test for age; chi-squared test for gender). The study estimates were extracted from the included papers according to the protocol below;

Study estimate extraction:

- Means and standard deviations were used as the basis for the study estimates, if reported.
- If these were not reported, then the median and interquartile ranges were extracted and converted into means and standard deviations using the methodology described in (45) and an online calculator (http://www.comp.hkbu.edu.hk/x~wan/median2mean.html).
- If the above estimates were not described in the text then they were estimated from the boxplots or graphs published in the text.

As all studies used different assays and units of measurement, it was not possible to do a direct comparison using the raw unstandardised mean difference. The study estimates were therefore used to calculate the standardized difference and the associated variance (yi and vi, respectively) using the metafor package for R in R studio (version 1.0.153), and the following formulas (44):

$$\begin{array}{l} yi=\frac{\bar{X}1-\bar{X}2}{Swithin} \end{array}$$

Where yi = standardized mean difference (*d*)

$\bar{X}1\;=$ sample mean in PD patients

$\bar{X}2\;=$ sample mean in controls

*Swithin* = within groups standard deviation, pooled across groups (as used above for the power calculation)

$$\begin{array}{l} Swithin=\;\sqrt{\frac{\left(n1-1\right){S1}^{2}+\left(n2-1\right){S2}^{2}}{n1+n2-2}} \end{array}$$

S1 = standard deviation in PD group

S2 = standard deviation in controls

A random effects model was used to assess the overall difference between patients and controls. Forest plots were generated to show the results graphically. Funnel plots were generated to plot standardized mean difference (*x* axis) against standard error (*y* axis) to assess the impact of publication bias and heterogeneity.

The variance of d (referred to as vi) is given by the following formula (see (44) page 27):

$$\begin{array}{l} vi=\frac{n1+n2}{n1n2}+\frac{d^{2}}{2\left(n1+n2\right)} \end{array}$$

## Results

A total of 17 papers met the inclusion and exclusion criteria for the systematic review (Table 1). Eight studies found a statistically significant increase in alpha synuclein antibodies in idiopathic PD patients compared to controls (30–33, 36, 37, 40, 42). These studies included a total of 305 patients and 198 controls but two of the papers appear to use overlapping patient samples with identical demographic tables and results figures and so the second of these was excluded (32, 42).

Three papers found raised alpha synuclein antibodies in sub-groups of PD patients, either in familial PD (35), pre-manifest LRRK2 carriers (39) or only in CSF and not serum (34). Four studies reported no difference in peripheral anti-alpha synuclein antibodies (9, 20, 38, 41) and two studies found that alpha synuclein antibodies were decreased in patients vs. controls (18, 19). Importantly the Brudek et al. paper focused on high affinity antibodies only which may underlie the difference in findings.

Three studies investigated antibodies in CSF as well as in plasma or serum (20, 31, 34) with two of these finding raised alpha synuclein antibodies in the CSF (31, 34).

All studies investigated the antibody response to full length alpha synuclein apart from the Caggiu et al study that assessed the response to specific epitopes deemed to be relevant due to their similarity to EBV (37).

### Clinical heterogeneity

There is wide variation in disease stage and duration across studies (see Table 1). Previous studies have noted an increase in early disease e.g., (42). Of the five papers reporting a mean disease duration of 5 years or less (see Table 1), four report an increase in alpha synuclein antibodies in patients compared to controls (representing a total of 196 patients and 121 controls excluding the first Gruden et al paper as described above) (9, 30, 31, 33, 36, 42). Only the smallest of the studies in early PD showed no PD-control difference (*N* = 14 PD patients and nine controls) (9). Even taking a conservative interpretation of these results, the larger studies are consistent in reporting an increase in alpha synuclein antibodies in early disease. An additional study for which disease duration was unavailable reported an association with HY disease stage with increasing titres from HY stage 1 to 2, decreasing at stage 3 (40). Alvarez-Castelao et al. found increased alpha synuclein antibodies in LRRK2 carriers vs. controls but not in patients with longer disease durations (>10 years) (39). Other studies have also reported a similar association with HY staging (33, 47). Of six studies with mean disease durations between 7 and 10 years, two studies report a clear increase in patients vs. controls (36, 37). Two further studies show an increase in a subgroup, in familial PD vs. controls (but not idiopathic PD) (35) in one study and in CSF only and not serum in another (34). The two studies that showed either no difference (38) or a difference in the opposite direction (19) did not have age and gender matched control groups. In the two studies with disease duration beyond 10 years there was either no difference (39) or a decrease in patients compared to controls (18).

Patient age also varies between study cohorts, ranging from a mean of 55.7 (36) to 69.8 [(37); Table 1]. Antibody responses vary with age and gender (47). It is therefore also critical to ensure that patient and control groups are well-matched. Of the 17 studies reviewed, seven either did not report appropriate demographic information or the control group was not matched to the patients.

### Assay variability

Most studies have made use of custom ELISAs with one study using a commercial ELISA for serum anti-alpha synuclein antibodies (33). Two positive studies by the same group in different patient cohorts used electroimpedence spectroscopy (30, 40). Several others used immunoblots or western blots (35, 36, 39). ELISAs are limited by many factors including the requirement for two independent binding events and problems with non-specific binding (30). There is also variation in conditions between studies, such as buffers used, protein coating concentration and temperature of the assay which are particularly relevant for an intrinsically disordered protein, such as alpha-synuclein.

Most of the alpha synuclein for the use in ELISAs was generated in *E. coli* in-house, and therefore may not include post-translational modifications present in mammalian cells (30, 31, 34–36, 38, 40) (with other papers obtaining commercially generated protein). Alvarez-Castelao et al. attempted to replicate their ELISA findings using immunoblots and identified that some of the ELISA positive samples were recognizing something other than alpha synuclein (39). This effect disappeared when they introduced an additional purification step suggesting the possibility that at least some of the findings in the literature may be due to interfering antibodies to bacterial toxins rather than to alpha synuclein itself. Antibodies present in serum may also be bound to serum protein (either specifically or non-specifically) which may interfere with antibody detection (38). Most of the papers investigated antibody responses to monomeric alpha synuclein (which is not necessarily the disease relevant species) with only a minority assessing responses to fibrils, mutated alpha synuclein (36, 39, 42), oligomers or other pathological forms [e.g., phosphorylated alpha synuclein (19) or specific peptides (37)]. The Brudek et al. paper focused on high affinity antibodies finding that these were decreased in patients compared to controls which is consistent with them having a role in alpha synuclein clearance. As other studies have investigated the overall antibody response it is not useful to directly compare these.

Lastly, some of the variation between studies may be due to the use of either serum or plasma (although only two studies used plasma rather than serum, see Table 1). It is possible that factors present in plasma but not in serum (e.g., alpha synuclein produced by platelets) may affect subsequent results and therefore it would be wise to standardize the use of serum across studies.

### Power

Lack of adequate power may be an important factor leading to false negative findings in a number of studies. The largest study included 93 PD patients and 194 controls (38) but unfortunately the controls were not age and gender matched to the patients (see Table 1). Of the 17 studies, seven included appropriate information to calculate power. Of those with incomplete information, this was usually because the data were presented as graphs or as medians and IQ range. The estimated sample sizes required to detect the differences reported ranged from 23 to 382, with a mean of 147 per group (see Table 1). The only study that was adequately powered was that by Gruden et al. that reported much larger difference between controls and patients than other studies and is therefore an outlier. Excluding this study, the estimated required sample size per group is between 60 and 382.

### Meta-analysis

**Figure 2:**
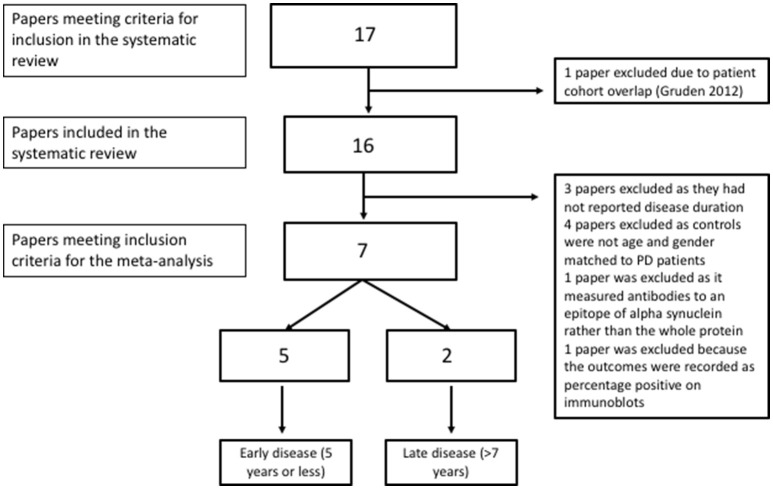
Flow diagram showing inclusion and exclusion of studies in the meta-analysis.

**Figure 3:**
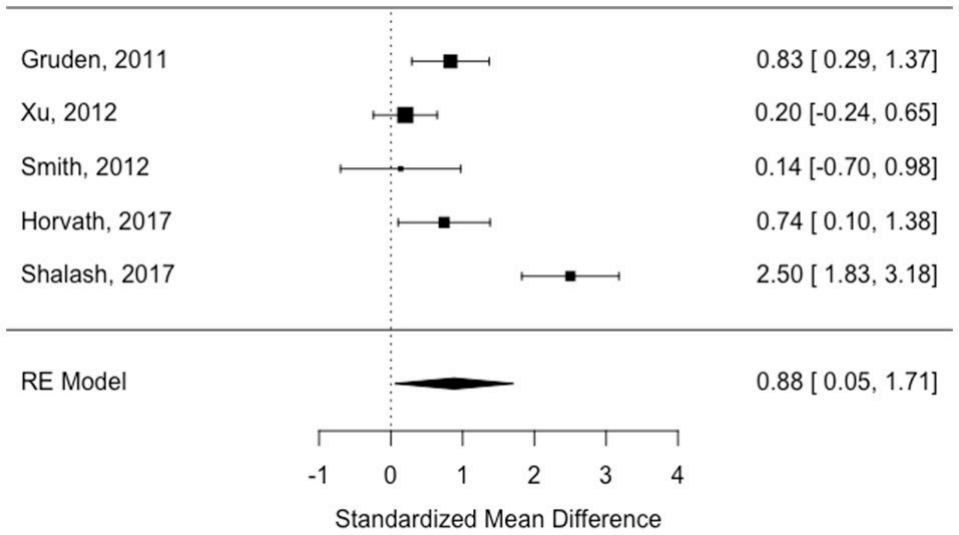
Forest plot showing study effect sizes in early disease (< 5.9 years).

All of the “early disease” papers shown in Table 1 met the inclusion criteria (see also flow plot in Figure 2) Means and standard deviations were available from two of the studies (30, 32). The means and standard deviations from Horvath et al. (31) were estimated based on the reported medians and interquartile ranges. The medians and interquartile ranges from the other two papers were estimated from boxplots and subsequently converted to means and standard deviations as described in the methods (9, 33). Study effect size estimates and model results are shown in Table 2. Overall, there is a significant increase in antibodies in patients vs. controls across studies (see forest plot in Figure 3) but the effect size is modest (0.88, 95% CI 0.05–1.71, *p*-value = 0.036). There was significant heterogeneity across studies (I^2^ = 89.32%).

**Table 2:** Study estimates, standardized effect sizes (yi) and variance (vi) (“early disease” < 5.9 years disease duration).

	**Year**	**Controls**			**PD**			**yi**	**vi**
		**Mean**	**SD**	**N**	**Mean**	**SD**	**N**		
Gruden	2011	25.00	50.99	26	310.00	452.55	32	0.83	0.08
Xu	2012	1.24	1.44	29	1.62	2.04	60	0.20	0.05
Smith	2012	0.83	1.13	9	1.06	1.81	14	0.14	0.18
Horvath	2017	5.00	0.67	20	6.50	2.72	20	0.74	0.11
Shalash	2017	0.49	0.69	20	4.39	1.78	46	2.50	0.12

*Model results: Estimate = 0.88 (95% CI 0.005–1.17), SE = 0.42, Z = 2.09, p = 0.036*.

*I^2^ = 89.32%*.

*Q(df = 4) = 33.71, p = 0.0001*.

Only two of the “later disease” studies (mean disease duration >7 years) met inclusion criteria (18, 36). Means and standard deviations were published in the Besong-Agbo study and therefore used to calculate study estimates. Medians and interquartile ranges were estimated from boxplots in the Yanamandra study which was divided into two subgroups (mean disease duration 6.7 years and mean disease duration 9.7 years as there was no available data for the patient group overall). Means and standard deviations were then derived from this data.

Three studies were excluded due to a lack of reported disease duration (20, 40, 41); four studies were excluded due to a lack of age and gender matching between patients and controls (19, 34, 38, 39). The Alvarez-Castelao paper did not include published significance testing of the age difference which was therefore done as part of this review. There was a significant difference in age between patients and controls according to a independent samples *t*-test (idiopathic PD mean 67.81, SD 9.98 and controls mean 61.4, SD 14.7), *t*[136] = 2.83, *p* = 0.005). One study was excluded as it only measured antibodies to specific epitopes of alpha synuclein rather than the entire protein (37) and one other study was excluded because outcomes were recorded as percentage positive on immunoblots (35).

**Figure 4:**
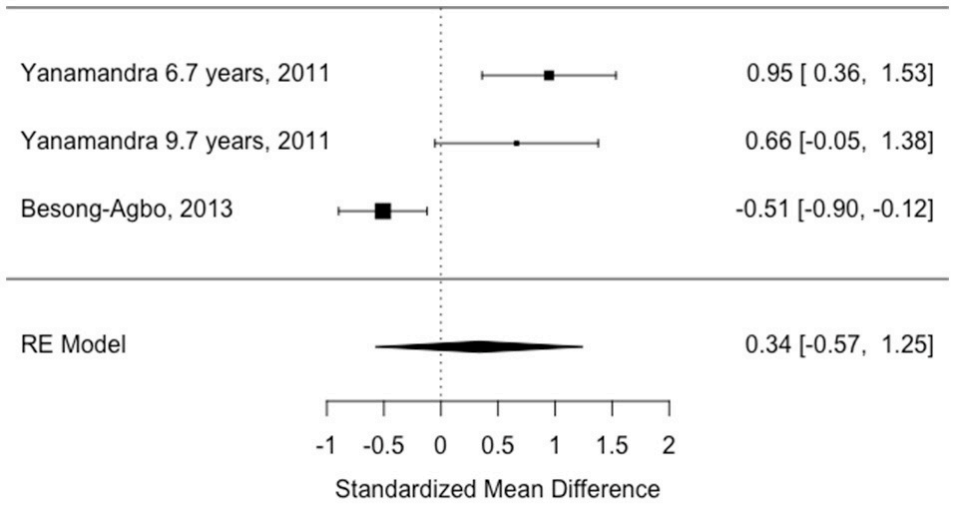
Forest plot showing study effect sizes in later disease (>7 years).

The study estimates are shown in Table 3 and the overall random effects model is shown in the forest plot in Figure 4. There was no overall difference between groups in this small sample (estimate = 0.34, 95% CI = −0.57–1.24, *p* = 0.46) and there was also significant heterogeneity (I^2^ = 87.67%).

**Table 3:** Study estimates, standardized effect sizes (yi) and variance (vi) (“later disease,” >7 years disease duration).

**Study**	**Year**	**Controls**			**PD**			**yi**	**vi**
		**Mean**	**SD**	**N**	**Mean**	**SD**	**N**		
Yanamandra 6.7 years	2011	108.67	126.43	23	696.44	821.82	27	0.95	0.09
Yanamandra 9.7 years	2011	108.67	126.43	23	313.11	490.58	12	0.66	0.13
Besong-Agbo	2013	153.5	103.77	46	105.40	85.83	62	−0.51	0.04

*Model results: Estimate 0.34 (95% CI −0.57–1.25), SE 0.46, Z = 0.73, p = 0.47*.

*I^2^ = 87.6%*.

*Q(df = 2) = 19.80, p = 0.0001*.

**Figure 5:**
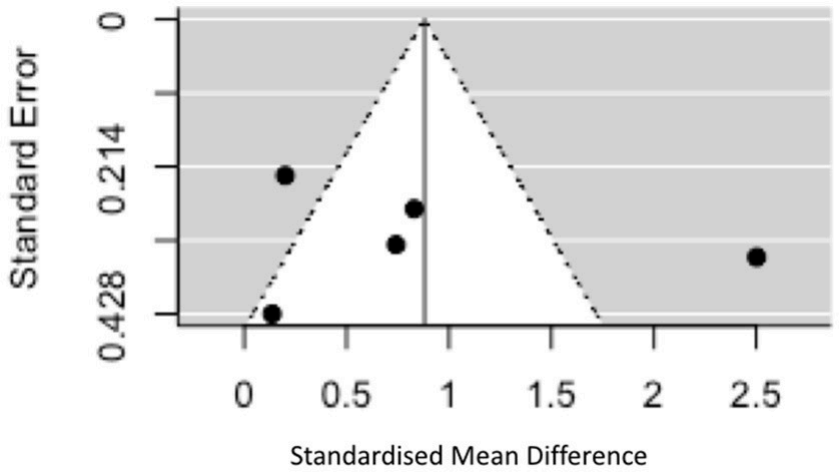
Funnel plot showing standard error vs. standardized mean difference in early disease.

Given the significant heterogeneity, funnel plots were generated plotting standardized mean difference on the *x* axis against standard error on the y axis for studies in the “early disease” group (there were too few in the later disease group to make interpretation of these plots meaningful). The plot is symmetrical around the effect size of 0.88 (*z* = 0.20, *p* = 0.84) but shows that two of the studies fall outside of the 95% CI of an assumed true effect (see Figure 5). One of the many explanations for the shape of this plot is the presence of true heterogeneity between studies (both clinical and assay related factors discussed above). If we were able to include more studies in the analysis one would expect, assuming the same true effect, that effect estimates from smaller studies would spread widely along the bottom with those from larger, more powerful studies appearing at the top (see Figure 5). One cannot fully discount the role played by publication bias in this context as positive findings in this field will be more likely to be written up and published than negative results particularly in the context of smaller studies.

## Conclusions and recommendations for future studies

Whilst the available data does not suggest elevation of alpha synuclein antibodies universally across all stages of PD, it is consistent with the hypothesis that there is an increased antibody response in early disease that wanes during disease progression, which is biologically plausible. According to our meta-analysis the effect size is modest in early disease but the analysis is limited by significant study heterogeneity.

There are many caveats to this conclusion based on both the systematic review and the meta-analysis, including the limitations of the assays used, clinical heterogeneity of cohorts, the lack of any longitudinal data and poor matching of controls to patient groups, meaning that the overall quality of evidence is poor (for example, seven of nine studies in later disease did not meet the inclusion criteria for the meta-analysis making it difficult to draw any firm conclusions from this aspect of the study). Hence the value of alpha synuclein auto-antibodies as a diagnostic or prognostic biomarker remains uncertain. Further studies are needed to demonstrate a consistent, reproducible effect in early PD cases vs. controls (or indeed between different groups of PD patients), to investigate the specificity of raised antibody titres in PD vs. other alpha-synucleinopathies, and to track longitudinal changes in antibody titres and their relationship to disease onset and clinical disease progression. The possible utility of using antibody based biomarkers for identifying patients who would potentially benefit from either immune modulating or antibody based therapies is also unknown.

There is a clear need for further studies in this field and we recommend that future studies should focus on the following points:

1. Appropriate sample size with an absolute minimum of 60 in each group (based on approximate power calculations from existing studies)
2. Well-characterized clinical cohorts with appropriately matched controls using both serum and CSF if possible
3. Longitudinal assessment to measure changes in antibody levels over the course of the disease and relationship with clinical disease progression
4. Study of prodromal PD cohorts to establish whether the antibody response is truly an early feature of the disease
5. Using a robustly validated method (ideally with validation using a second method in the same samples) to measure antibodies including standardization and testing of different coating concentrations, buffers and assay temperature.
6. Study of epitope-specific antibodies and Ig subclasses to allow a fuller understanding of the adaptive immune response to PD.

## Author contributions

KS designed the study, reviewed the literature, performed the meta-analysis and wrote the first draft of the manuscript. SY and AK reviewed the literature, made summary tables and critically reviewed the manuscript. MC and CW-G contributed significantly to the design of the study and critically reviewed the final manuscript.

### Conflict of interest statement

The authors declare that the research was conducted in the absence of any commercial or financial relationships that could be construed as a potential conflict of interest.
